# *E*ffect of *A R*eduction in glomerular filtration rate after *NE*phrectomy on arterial *ST*iffness and central hemodynamics: Rationale and design of the EARNEST study^☆^

**DOI:** 10.1016/j.ahj.2013.10.024

**Published:** 2014-02

**Authors:** William E. Moody, Laurie A. Tomlinson, Charles J. Ferro, Richard P. Steeds, Patrick B. Mark, Daniel Zehnder, Charles R. Tomson, John R. Cockcroft, Ian B. Wilkinson, Jonathan N. Townend

**Affiliations:** a Birmingham Cardio-Renal Group, Centre for Clinical Cardiovascular Science, Nuffield House, Queen Elizabeth Hospital Birmingham, Birmingham, United Kingdom; b Cambridge Clinical Trials Unit, Clinical School, Addenbrooke's Hospital, University of Cambridge, Cambridge, United Kingdom; c BHF Cardiovascular Research Centre, Institute of Cardiovascular and Medical Sciences, University of Glasgow, Glasgow, United Kingdom; d Clinical Science Research Laboratory, University of Warwick, Coventry, United Kingdom; e Academic Renal Unit, Learning and Research, Southmead Hospital, Westbury-on-Trym, Bristol, United Kingdom; f Department of Cardiology, Wales Heart Research Institute, University Hospital, Cardiff, United Kingdom

## Abstract

**Background:**

There is strong evidence of an association between chronic kidney disease (CKD) and cardiovascular disease. To date, however, proof that a reduction in glomerular filtration rate (GFR) is a causative factor in cardiovascular disease is lacking. Kidney donors comprise a highly screened population without risk factors such as diabetes and inflammation, which invariably confound the association between CKD and cardiovascular disease. There is strong evidence that increased arterial stiffness and left ventricular hypertrophy and fibrosis, rather than atherosclerotic disease, mediate the adverse cardiovascular effects of CKD. The expanding practice of live kidney donation provides a unique opportunity to study the cardiovascular effects of an isolated reduction in GFR in a prospective fashion. At the same time, the proposed study will address ongoing safety concerns that persist because most longitudinal outcome studies have been undertaken at single centers and compared donor cohorts with an inappropriately selected control group.

**Hypotheses:**

The reduction in GFR accompanying uninephrectomy causes (1) a pressure-independent increase in aortic stiffness (aortic pulse wave velocity) and (2) an increase in peripheral and central blood pressure.

**Methods:**

This is a prospective, multicenter, longitudinal, parallel group study of 440 living kidney donors and 440 healthy controls. All controls will be eligible for living kidney donation using current UK transplant criteria. Investigations will be performed at baseline and repeated at 12 months in the first instance. These include measurement of arterial stiffness using applanation tonometry to determine pulse wave velocity and pulse wave analysis, office blood pressure, 24-hour ambulatory blood pressure monitoring, and a series of biomarkers for cardiovascular and bone mineral disease.

**Conclusions:**

These data will prove valuable by characterizing the direction of causality between cardiovascular and renal disease. This should help inform whether targeting reduced GFR alongside more traditional cardiovascular risk factors is warranted. In addition, this study will contribute important safety data on living kidney donors by providing a longitudinal assessment of well-validated surrogate markers of cardiovascular disease, namely, blood pressure and arterial stiffness. If any adverse effects are detected, these may be potentially reversed with the early introduction of targeted therapy. This should ensure that kidney donors do not come to long-term harm and thereby preserve the ongoing expansion of the living donor transplant program (NCT01769924).

## Background and rationale

The robust epidemiologic evidence that shows chronic kidney disease (CKD), as manifest by reduced estimated glomerular filtration rate (eGFR), is associated with increased cardiovascular morbidity and mortality provides a strong rationale for a prospective study to determine the cardiovascular effects of kidney donation. A graded inverse relationship exists between cardiovascular risk and eGFR^1^; deleterious cardiovascular effects are clearly evident once eGFR falls to <60 mL/min per 1.73 m^2^, although the exact threshold at which cardiovascular risk becomes elevated remains contentious.^2^, ^3^ Although most data indicate that the cutoff lies between 60 and 70 mL/min per 1.73 m^2^, it may be even higher; in a meta-analysis of general population cohorts with a median follow-up of about 8 years and more than 5 million person-years, mortality rose exponentially once eGFR fell below 75 mL/min per 1.73 m^2^.

Every year, more than 6,000 US individuals undergo elective nephrectomy for the purposes of live donation without apparent increased long-term mortality or cardiovascular risk.^4^, ^5^ Uninephrectomy may be qualified as an “acute kidney injury,” with an immediate 50% reduction in GFR followed by an improvement related to hypertrophy in the remaining kidney—but only to 60% to 70% of baseline.^6^ Up to two-thirds of donors after nephrectomy fulfil the criteria for CKD stage 3 (eGFR, 30-59 mL/min per 1.73 m^2^) dependent on baseline age and renal function,^7^ which has an approximate odds ratio for cardiovascular disease of between 2 and 4 in the general population.^8^ To date, however, all studies examining the long-term consequences of kidney donation have been reassuring, often showing better health outcomes in donors compared with the general population.^4^, ^9^, ^10^ Small adverse cardiovascular effects of donation cannot be excluded, however, because of residual confounding from selection and follow-up biases. Most donors are highly motivated individuals, often making healthy lifestyle choices both before and after nephrectomy. This inherent altruistic nature, in conjunction with the rigorous medical selection procedures for donors, results in an extreme low-risk population, for whom it is near impossible to identify a control cohort of equivalent health status from the general population. This makes well-controlled studies difficult, and thus, far we are aware of only one published prospective, controlled pathophysiologic study of kidney donors.^11^

The mechanisms by which CKD exerts adverse effects on cardiovascular structure and function are diverse and have been the subject of recent reviews.^12^, ^13^ Leading potential mediators include increased arterial stiffness, hypertension, increased left ventricular (LV) mass and abnormal bone mineral metabolism, which may lead to increased vascular calcification.^12^ There is strong cross-sectional evidence that aortic pulse wave velocity (aPWV; a validated measure of arterial stiffness) increases as GFR falls.^14^, ^15^, ^16^ In a community-based study, among treated hypertensive patients, even minimally impaired baseline renal function was associated with an increased rate of aortic stiffening.^17^ Aortic PWV is an independent predictor of all-cause and cardiovascular mortality in many populations including patients on dialysis,^18^, ^19^ and failure to lower aPWV by angiotensin-converting enzyme inhibitors and blood pressure reduction has been associated with reduced survival.^20^ To date, only one study examining arterial stiffness in kidney donors after nephrectomy has been published.^21^ This had a cross-sectional observational design, comparing 101 kidney donors at a mean of 111 ± 42 months from donation with healthy controls matched only for age and gender, indicating that aPWV was significantly higher in donors. In a recent uncontrolled study of 17 donors, although aPWV was not examined, augmentation index and central arterial pressure were unchanged at 6 months compared with baseline.^22^

With respect to the effects of kidney donation on peripheral blood pressure, the data are inconclusive, but the best available evidence from a meta-analysis suggests a moderate increase of about 5 mm Hg in systolic pressure at 10 years after donation.^23^ Although high-quality prospective studies of donors are still required, in the general population, even a 2-mm Hg increase in systolic blood pressure is associated with a long-term increase in mortality of about 10% for stroke and 7% for ischemic heart disease.^24^ Thus, it appears that either the adverse effects of hypertension in kidney donors are small and as yet undetected by clinical end point studies or that regular medical supervision postdonation has allowed early initiation of antihypertensive therapy and thereby prevented harmful sequelae.

Increased LV mass is an almost universal finding by late-stage CKD^25^ and is recognized as an important mediator of morbidity and mortality due to heart failure and lethal arrhythmia, the commonest modes of death in CKD.^26^ Chronic Kidney Disease–Mineral and Bone Disorder (CKD-MBD) is driven by impaired phosphate excretion resulting in both bone disease and vascular calcification, which, in turn, mediates increased arterial stiffness and adverse LV remodeling.^27^, ^28^ Markers of CKD-MBD such as phosphate, fibroblast growth factor 23 (FGF-23), and parathyroid hormone (PTH) predict mortality and adverse cardiovascular events in patients with CKD.^29^ Furthermore, there is evidence from community-based studies of graded associations between phosphate and LV mass, cardiovascular events, and death.^30^ Further detailed information is therefore required on the effect of kidney donation on both LV mass and markers of CKD-MBD. A 23% increase in PTH has already been reported in kidney donors at 6 months after nephrectomy compared with controls.^11^

Although reduced eGFR is strongly associated with increased cardiovascular risk, this relationship may not be causal: CKD has a powerful clustering effect for other cardiovascular risk factors such as hypertension, diabetes, dyslipidemia, and inflammation, which may confound the association. Kidney donors provide an ideal “human model” that enables the longitudinal study of the cardiovascular effects of a reduction in GFR in isolation without the multiple confounding factors associated with renal disease. The proposed study will prospectively examine the effects of unilateral nephrectomy on the potential pathophysiologic mechanisms that appear to mediate cardiovascular morbidity and mortality in CKD, namely, arterial stiffness and blood pressure as primary end points, and LV mass and CKD-MBD parameters as nested substudies, in a cohort of kidney donors large enough to allow the detection of small effect sizes.

### Hypotheses

In living kidney donors, the reduction in GFR that accompanies nephrectomy results in (1) a pressure-independent increase in aortic stiffness (aPWV), (2) an increase in blood pressure, (3) an increase in LV mass and impairment in LV diastolic function, and (4) adverse effects on bone mineral metabolism.

## Methods

### Study design

This is a prospective, multicenter, longitudinal, parallel group study of 440 living kidney donors and 440 healthy controls to be recruited for 2 years with initial follow-up of 12 months. All participants will undergo informed written consent in keeping with the principles set out by the Declaration of Helsinki. The EARNEST study received research ethics approval in February 2013. The study is funded by the British Heart Foundation and registered with the US National Institutes of Health database (NCT01769924).

### Study population

The inclusion and exclusion criteria for both donor subjects and controls are outlined in Table I and are identical to those set by the UK living kidney donor guidelines compiled by the Joint Working Party of The British Transplantation Society and The Renal Association.^31^ The only inclusion criterion is that donor subjects will be scheduled for nephrectomy for the purpose of kidney donation. A carefully matched series of control patients will be recruited from the same living donor clinics at which subjects are identified, who, after screening, are found to be fit for donation but do not proceed to surgery (eg, because of arterial anatomy and immunologic mismatch). If appropriate, donor-related family members will also be invited to participate in the study as healthy controls to provide a closely matched control population with equivalent baseline health status to the donor cohort.

**Table I:** Inclusion and exclusion criteria

Inclusion criteria
Age 18-80 y
Acceptable GFR by donor age before donation⁎
Exclusion criteria
Hypertensive end-organ damage, uncontrolled hypertension or the requirement for >2 antihypertensive medications
Significant proteinuria†
LV dysfunction
Diabetes mellitus
Atrial fibrillation
Any history of cardiovascular or pulmonary disease that would preclude kidney donation

⁎ Based on the anticipation of having an eGFR of >50 mL/min per 1.73 m^2^ aged 70 years.

† ACR >30 mg/mmol, PCR >50 mg/mmol, or 24-hour total protein >300 mg/d.

Participants will be recruited from the following core centers, chosen because of their high numbers of live donor transplants and strength in vascular research: (1) Queen Elizabeth Hospital Birmingham, England; (2) Addenbrooke's Hospital, Cambridge, England; (3) Western Infirmary Glasgow, England; (4) University Hospital Coventry and Warwick, England; (5) Southmead Hospital, Bristol, England; (6) Northern General Hospital, Sheffield, England; and (7) Belfast City Hospital, Belfast, North Ireland. This study remains open to the inclusion of additional transplant centers from inside and outside the UK.

### Study protocol

A flowchart of the study protocol is presented in the Figure. Investigations will be performed in all participants at baseline in controls and within the 6 weeks before nephrectomy in donors, with subsequent follow-up studies performed at 12 months. Kidney donors will undergo routine follow-up by their local medical and surgical team with no alteration to normal care. No restrictions will be made to the introduction of any treatment including antihypertensive drugs. At baseline and follow-up visits, body mass index (BMI), blood pressure, heart rate, and any clinical events will be recorded.

**Figure:**
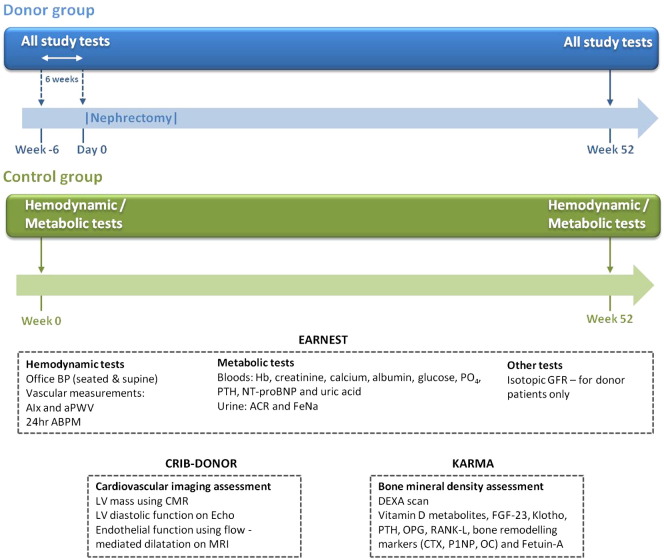
EARNEST study timeline. Abbreviations: ABPM, ambulatory blood pressure monitoring; AIx, augmentation index; BP, blood pressure; CTX, carboxy-terminal collagen cross-links; FeNa, fractional excretion of urinary sodium; Hb, hemoglobin; MRI, magnetic resonance imaging; NT-proBNP, N-terminal prohormone of brain natriuretic peptide; OC, osteocalcin; OPG, osteoprotegerin; PO_4_, phosphate; P1NP, N-terminal propeptides of type I procollagen; RANK-L, receptor activator of nuclear factor κB ligand.

Table II summarizes the main outcome variables of interest and the relevant data collection methods. Office blood pressure will be measured on 3 occasions using a validated automated device. The first measurement will be made after at least 5 minutes of seated rest. Two further readings will be taken with at least 1 minute between readings. All measurements will be made from the nondominant arm using the same cuff size. Participants will also undergo 24-hour ambulatory blood pressure monitoring using a British Hypertension Society validated oscillometric recorder (Mobil-O-Graph NG; IEM, Stolberg, Germany), which will provide data on both peripheral and central blood pressure.^32^, ^33^

**Table II:** Summary of EARNEST outcome variables and data collection methods

Outcome	Method
Primary outcomes	
Aortic stiffness (aPWV)	aPWV (SphygmoCor; AtCor Medical) will be performed after 15 min of supine rest using a high-fidelity micromanometer (SPC-301; Millar Instruments), as previously described.^34^ Path length is taken as sternal notch-to-femoral pulse distance minus sternal notch-to-carotid pulse distance.
Mean 24-h ambulatory peripheral and central systolic blood pressure	Mean systolic blood pressure from the nondominant arm using a 24-h monitoring system (Mobil-O-Graph NG; IEM). Readings taken every 30 min during the day (0800-2200) and every 60 min overnight
Secondary outcomes	
Office systolic and diastolic blood pressure	Three separate measurements taken after at least 5 min of seated rest using a BHS validated automated device, mean of last 2 recorded
Other ambulatory blood pressure parameters	Mean 24 h and daytime systolic and diastolic blood pressure, pulse pressure
New-onset hypertension	Diagnosis is defined as new antihypertensive therapy and/or systolic blood pressure >135 mm Hg or diastolic blood pressure >85 mm Hg during the daytime on 24-h ambulatory monitoring.
Augmentation index and central hemodynamics	A micromanometer (SPC-301; Millar Instruments) will be used to flatten but not occlude the radial artery using gentle pressure. An averaged peripheral waveform and corresponding central waveform will be generated after 11 s of data capture. The central waveform will then be analyzed to determine augmentation index and central aortic pressures.
Renal function	Isotopic GFR using the renal clearance of ^51^Cr-EDTA will be measured in donors.
Bloods	Hemoglobin, creatinine, calcium, albumin, glucose, phosphate, PTH, NT-proBNP, and uric acid
Urine	ACR and fractional excretion of sodium

Abbreviations: *BHS*, British Hypertension Society; *EDTA*, ethylenediaminetetraacetic acid; *NT-proBNP*, N-terminal prohormone of brain natriuretic peptide.

#### Pulse wave velocity

Aortic PWV is the current criterion standard technique for measuring aortic stiffness.^34^ Subjects will be studied in a quiet, temperature-controlled laboratory after 15 minutes of lying supine. Pulse wave velocity (SphygmoCor; AtCor Medical, Sydney, Australia) will be determined by sequential acquisition of pressure waveforms from the carotid and femoral arteries by use of a high-fidelity micromanometer (SPC-301; Millar Instruments, Houston, TX), as previously described.^35^, ^36^ The path length will be calculated by subtracting the distance between the sternal notch and the carotid recording site from the distance between the sternal notch and the femoral site.

#### Pulse wave analysis

Blood pressure measurement will be repeated supine before undertaking applanation tonometry to record high-fidelity arterial pressure waveforms from which indices relating to large artery stiffness can be calculated. Central pressure waveforms will be derived and analyzed using the technique of pulse wave analysis, as previously described.^35^, ^37^ A micromanometer will be used to flatten but not occlude the radial artery using gentle pressure. An averaged peripheral waveform and corresponding central waveform will be generated after 11 seconds of data capture. The central waveform will then be analyzed to determine the augmentation index (the difference between the second and first peaks of the central pressure waveform, expressed as a percentage of the pulse pressure) and central aortic pressures. The method has been shown to be reproducible in both healthy subjects and in patients with CKD. Augmentation index and aPWV will also be recorded using ambulatory measurements taken from the Mobilograph.^38^, ^39^

#### Training and reproducibility

Delegated study staff will receive formal training in measurement techniques of arterial stiffness and undertake a reproducibility and measurement quality study before study commencement. Measurements at each center will only be performed by dedicated staff who have undergone this training. Equipment will be calibrated at baseline and throughout the study at specific intervals.

#### Assessment of renal function

Isotopic GFR for kidney donors will also be measured using the renal clearance of ^51^Cr-EDTA.^40^ Kidney function in controls will be estimated from serum creatinine measurement.^41^, ^42^

#### Blood and urine

Routine hematologic and biochemical parameters will be recorded, including hemoglobin, creatinine, calcium, albumin, glucose, phosphate, and PTH. A series of biomarkers will also be measured including N-terminal prohormone of brain natriuretic peptide, uric acid, urinary albumin/creatinine ratio (ACR), and fractional excretion of sodium.^43^, ^44^, ^45^

#### Long-term follow-up

Patients will also be asked to consent to longitudinal follow-up using the NHS Health & Social Care Information Centre's Data Linkage Service, which provides the patient status and tracking service to examine long-term outcomes after the study ends.

The authors are solely responsible for the design and conduct of this study, all study analyses, the drafting and editing of the manuscript, and its final contents.

## EARNEST substudies

### The Chronic Renal Impairment in Birmingham–Kidney Donor (CRIB-DONOR) study: a cardiac magnetic resonance (CMR) and echocardiography study

Those participants enrolled into the study at Queen Elizabeth Hospital Birmingham and some other centers will undergo CMR imaging and echocardiography at baseline and 12 months to determine the effects of an isolated reduction in GFR on LV mass and function as well as aortic compliance and endothelial function (online Supplementary Table I). Arterial stiffness and office and ambulatory blood pressure will also be measured at 6 months (in addition to the baseline and 12-month measurement required for EARNEST). This study is registered separately with the US National Institutes of Health database (NCT01028703) and has a current recruitment target of 140 participants.

#### Cardiac magnetic resonance imaging

Cardiac magnetic resonance imaging will be performed using a 1.5-T scanner (Magnetom Avanto; Siemens, Erlangen, Germany). Serial contiguous short-axis cines will be piloted from the vertical long-axis and horizontal long-axis images of the left and right ventricles (electrocardiogram R-wave gated, steady-state free precession imaging [True-FISP]; temporal resolution 40-50 ms, repetition time 3.2 ms, echo time 1.7 ms, flip angle 60°, slice thickness 7 mm with 3-mm gap) in accordance with previously validated methodology.^46^ Analysis will be performed offline (Argus Software; Siemens) by a single blinded observer for the assessment of ventricular volumes (end-diastolic, end-systolic and stroke volumes), function (ejection fraction), and LV mass.^46^, ^47^ In the determination of ventricular mass, volumes, and function, as compared with transthoracic echocardiography, CMR has higher interstudy reproducibility, thereby reducing the number of participants required to adequately power such studies.^48^ Aortic distensibility will be assessed at the ascending aorta and proximal descending aorta at the level of the pulmonary artery and the distal descending aorta at the level of the diaphragm on held end-expiration and calculated using previously validated formulae.^49^ Measurement of aortic distensibility using CMR has low intraobserver variability and good reproducibility.^50^ Peripheral blood pressure will be performed synchronously, in triplicate, at the brachial artery at the time of scanning for determination of pulse pressure.

Dynamic tissue-tagging MRI allows direct noninvasive assessment of regional systolic and diastolic function and is a previously validated technique.^51^ Spatial modulation of magnetization will be used to generate a uniform grid pattern with 8-mm tag separation on the LV myocardium at 3 short-axis sections (basal, mid, and apex) and the horizontal long-axis image using a fast filed echo multishot sequence (temporal resolution 40-50 ms, repetition time 3.9 ms, echo time 4.4 ms, voxel size 1.8/1.3/6.0 mm^3^, flip angel 14°, tag grid angle 45° with slice thickness 6 mm, and a minimum number of 15 phases per cardiac cycle) with prospective electrocardiogram gating, as previously described.^52^ Blinded analysis will be performed offline (CIMTag2D; University of Auckland, New Zealand) for LV strain, strain rate, and torsion calculation, as previously described.^52^

Flow-mediated dilatation of the brachial artery will also be measured using magnetic resonance measurement of brachial artery area before and after 5 minutes of arterial occlusion and also after 400-μg glyceryl trinitrate (GTN) control.^53^ Brachial artery measurements will be performed offline using semiautomated contour tracking software (MATLAB R2008b; MathWorks Inc, Natick, MA) calculating flow-mediated dilatation as the proportional change in artery diameter in response to hyperemia.^54^

#### Transthoracic echocardiography

A comprehensive transthoracic echocardiogram (iE33; Philips, Eindhoven, the Netherlands) will be performed at rest with the subject in the left lateral decubitus position by a single experienced sonographer using second harmonic imaging and an S5-1 multifrequency transducer. All parameters will be measured in triplicate and averaged as per the American Society of Echocardiography guidelines.^55^ Analysis will be performed offline by a single blinded observer on an Xcelera workstation (Philips). Ventricular dimensions, wall thickness, chamber volumes, and stroke volume will be determined using standard methods.^56^ Left ventricular diastolic function will be determined using standard techniques.^57^ Peak systolic (*s*′), early diastolic (*e*′), and late diastolic (*a*′) mitral annular velocities will be measured at end-expiration at the septal, lateral, inferior, and anterior LV walls with real-time pulsed wave tissue Doppler imaging (TDI).^58^

Gray-scale images for 2-dimensional LV strain, strain rate, and torsion will be acquired in cineloop format in triplicate from the apical 4-, 2-, and 3-chamber views and parasternal short-axis views at basal, mid, and apical levels at end expiration at frame rates >70 Hz for offline analysis using commercially available software (QLAB; Philips). Myocardial contours will be manually traced at end-systole, and the software will then generate a region of interest over the myocardium. This will enable frame-to-frame tracking of ultrasonic speckles that change position according to surrounding tissue motion throughout the cardiac cycle. Peak systolic velocities, strain, strain rate, rotation, and twist will be measured for each myocardial segment in triplicate and averaged.

In those subjects with appropriate image quality, transthoracic echocardiography will also be performed during a progressive submaximal exercise test to derive stress-related changes in LV systolic and diastolic function using TDI and strain measures. Subjects will be installed on a dedicated semisupine cycling ergometer (Schiller ERG 911 BP/L, Baar, Switzerland). After a 15-minute rest period, each subject will undergo an exercise test that includes 3 workload stages of 4-minute duration at 20%, 30%, and 40% of their maximal aerobic power estimated via the Wasserman equation [(body mass (50.72 − 0.372 × age)) − 350]/10.3 and corrected for semisupine position (20% removed from normal values).^59^, ^60^

### The KARMA study: effect of *K*idney don*A*tion on bone-mine*R*al *M*etabolism and *A*rterial stiffness

Participants enrolled into the study at Addenbrooke's Hospital, Cambridge, will undergo a more detailed biochemical assessment of the bone mineral axis together with dual-energy x-ray absorptiometry (DEXA). It is planned to recruit 99 participants for the KARMA study, 2 controls for every living donor (online Supplementary Table II).

#### Dual-energy x-ray absorptiometry scan

Dual-energy x-ray absorptiometry is a means of measuring bone mineral density and soft tissue composition. A beam of x-ray, filtered into 2 energy bands, passes through the patient's body. When soft tissue absorption is subtracted out, the bone mineral density can be determined from the absorption by bone at each energy. Dual-energy x-ray absorptiometry is the most widely used and most thoroughly studied bone density measurement technology. For kidney donors, DEXA scans of the total body, hips, and lumbar spine scanning will be performed before surgery and 1 year later. The total body scan will enable any changes in muscle mass to be estimated and their influence on bone density factored out of the bone density analysis. The total effective dose of x-ray radiation for the proposed scans will be approximately 4.5 μSv, comparable with a daily background from atmospheric sources of 6 μSv. Pregnancy testing (urinary β-human chorionic gonadotropin) will be performed in female participants of childbearing age in the donor group before DEXA scanning.

### Blood tests

These will be the same as for the main EARNEST study, but for kidney donors, additional samples will be taken at days 1, 2, and 3 postoperatively and 6 weeks after donation. Blood samples will be centrifuged within 30 minutes of venesection, and serum and plasma were separated and frozen at −70°C. Samples will later also be used for testing of vitamin D metabolites (1, 25-, and 25-vitamin D), FGF-23, Klotho, PTH, osteoprotegerin, receptor activator of nuclear factor κB ligand, bone remodeling markers (carboxy-terminal collagen cross-links, N-terminal propeptides of type I procollagen, osteocalcin), and Fetuin-A as well as renin and aldosterone.

#### Urine tests

Again, these will be the same as for the main EARNEST study, but in addition for kidney donors, 100 mL aliquots of urine will be taken at days 1, 2, and 3 postoperatively and 6 weeks after surgery and frozen at −70°C for later measurement of Fetuin-A, calcium, and phosphate.

### Statistical analysis

The formal comparison between arms of each of the coprimary end points will use a linear regression model on the within-patient change from baseline, adjusting for baseline value, sex, ethnicity, use of antihypertensives, age, eGFR at donation (and iGFR where available in donors), and the other coprimary end point at baseline. To formally control the overall significance level over both coprimary end points (aPWV and blood pressure) at 5%, a Holm-Bonferroni method will be used. Findings will be interpreted taking into consideration that aPWV and blood pressure are correlated. A secondary analysis of the coprimary end points will be performed according to age, ethnic group, baseline eGFR and change in eGFR (and isotopic GFR where available in donors), BMI, and predonation hypertension.

### EARNEST power calculations and sample size

Assuming that the SD of the within-patient change is 1.0 m/s in aPWV and 10 mm Hg in blood pressure,^35^, ^61^ a sample size of 800 patients (control and donors, 400 subjects each, assuming 9% dropout) will provide 80% power to detect a difference of 0.22 m/s or 2.2 mm Hg for aPWV and blood pressure, respectively, under a 2-sided *t* test at the 2.5% significance level. Power calculations for CRIB-DONOR and KARMA substudies are outlined in the online Appendix.

## Discussion

There is strong evidence that patients with CKD have increased cardiovascular risk with a graded relation with both eGFR and the level of albuminuria.^62^ However, previous studies are limited by their cross-sectional and observational nature, and causation has not yet been established. The true impact of CKD on the cardiovascular system remains hard to define because the duration of the CKD is often unknown, the underlying causes are diverse, and there are numerous confounders including associated diseases such as hypertension and diabetes. Moreover, the level of GFR at which adverse structural and functional changes occur within the heart and vasculature and at which cardiovascular risk starts to rise is not clear. Kidney donors provide a unique healthy population who change at a known time point from having normal to impaired renal function. This study should establish whether reduced GFR leads directly to adverse cardiovascular effects such as increased arterial stiffness, hypertension, LV mass, and mineral bone disorder. Because the prevalence of CKD is high and rising (14.0% in the United States in 2005-2010, as defined by eGFR <60 mL/min per 1.72 m^2^ or ACR ≥30 mg/g),^26^ it is important to establish if a causal relationship exists to determine whether targeting CKD alongside more traditional risk factors could lead to reductions in cardiovascular mortality and morbidity. The potential size of this effect is salutary. If causative, it has been estimated that up to 10% of vascular events in middle age and 20% in old age might be attributable to reduced eGFR.^63^

By careful prospective measurement of ambulatory blood pressure in living kidney donors before and after nephrectomy, this study will also provide important practical information needed for patient care. By clarifying whether there is an increased frequency of hypertension and surrogate markers of cardiovascular disease after donation, we will be able to better advise potential donors in future and inform national guidance on the importance of long-term follow-up.

In an attempt to reduce the shortfall in the number of organs available for transplantation, the opportunity to undergo donor nephrectomy is now being offered to people with preexisting medical conditions, which include hypertension and raised BMI, as well as to subjects who are older with lower baseline GFR. The potential renal and cardiovascular risks of kidney donation may be most relevant to this group of donors, but those studies that have attempted to address this issue are single-center, retrospective reports offering conflicting data.^64^, ^65^, ^66^ There may also be increased risk in nonwhite donors, although the data in this area remain preliminary.^9^ The EARNEST study is large enough to prospectively examine the influences of age, BMI, ethnicity, and comorbidity on changes in cardiovascular structure and function after nephrectomy in kidney donors.

We believe that the living kidney donation program is a vital method of providing renal replacement therapy for individuals with end-stage kidney disease. The evidence to date suggests that this practice is safe, although there are data demonstrating an increased risk of high blood pressure after donation. Studying vascular parameters in detail both before and after donation provides an excellent opportunity to examine prospectively the direction of causality between loss of kidney function and cardiovascular disease. In addition, we may be able to provide important information to guide the long-term management of kidney donors. An open, critical approach is vital if we are to preserve public trust and the altruistic goodwill of donors, with the aim of safeguarding the expansion of the living kidney transplant program.

## Disclosures

The EARNEST study is funded by a British Heart Foundation Project Grant. W.E.M. is supported by a British Heart Clinical Research Fellowship. We are also grateful for the resources provided by the Comprehensive Local Research Networks (CLRN), the Wellcome/National Institute for Health Research (NIHR) Clinical Research Facilities in Birmingham and Cambridge, and The British Heart Foundation Research Centre in Glasgow.

## Figures and Tables

**Supplementary Table I:** Summary of CRIB-DONOR study main outcome variables and data collection methods

Outcome	Method
Primary outcomes	
LV mass	Images acquired using 1.5-T CMR imaging (Magnetom Avanto; Siemens). Analysis will be performed offline (Argus Software; Siemens) by a single blinded observer.
Aortic stiffness (aPWV)	As for EARNEST, see Table II.
Secondary outcomes	
New-onset hypertension	As for EARNEST, see Table II.
Augmentation index and central hemodynamics	As for EARNEST, see Table II.
LV systolic and diastolic function	Myocardial tagging (SPAMM) will be performed on magnetic resonance images and analysis performed offline by a single blinded observer (CIMTag2D; University of Auckland).
	Transthoracic echocardiography will be undertaken (iE33; Philips) and analysis performed offline by a single blinded observer on an Xcelera workstation (Philips).
Endothelial function	Magnetic resonance imaging (Magnetom Avanto; Siemens) to calculate flow-mediated dilatation as the proportional change in artery diameter in response to hyperemia. Analysis will be performed offline using semiautomated contour tracking software (MATLAB R2008b; MathWorks Inc).
Exercise-induced changes in LV strain and diastolic function	Progressive submaximal exercise testing to derive stress-related changes in LV systolic and diastolic function using TDI and strain measures. Subjects will be installed on a dedicated semisupine cycling ergometer (Schiller ERG 911 BP/L).
Bloods	As for EARNEST, see Table II.
	In addition: biomarkers of oxidative stress, endothelial function, collagen turnover
Urine	As for EARNEST, see Table II.

*SPAMM*, Spatial modulation of magnetization imaging.

**Supplementary Table II:** Summary of KARMA study main outcome variables and data collection methods

Outcome	Method
Aortic stiffness (aPWV)	As for EARNEST, see Table II.
New-onset hypertension	As for EARNEST, see Table II.
Augmentation index and central hemodynamics	As for EARNEST, see Table II.
Bone mineral density	Kidney donor participants will undergo DEXA scanning (Lunar Prodigy Advance; GE Healthcare, Bedford, UK) of the total body, hips, and lumbar spine at baseline and 1 y. The total body scan will enable any changes in muscle mass to be estimated and their influence factored out of the bone density analysis.
Bloods	As for EARNEST, see Table II.
	In addition: vitamin D metabolites (1, 25-, and 25-vitamin D), FGF-23, Klotho, PTH, OPG, RANK-L, bone remodeling markers (CTX, P1NP, OC), and Fetuin-A
Urine	As for EARNEST, see Table II.
	In addition: Fetuin-A, fractional excretion of calcium, phosphate

Abbreviations: *OPG*, Osteoprotegerin; *RANK-L*, receptor activator of nuclear factor κB ligand; *CTX*, carboxy-terminal collagen cross-links; *P1NP*, N-terminal propeptides of type I procollagen; *OC*, osteocalcin.

## Appendix

### CRIB-DONOR power calculations and sample size

The major end point on which sample size and power for the CRIB-DONOR study have been calculated is LV mass. Assuming an SD of the within-patient change in LV mass index of 12 g/m^2^,^67^ a sample size of 63 subjects per group will provide >95% power to detect a change in LV mass index of 7.5 g/m^2^ with an *α* value of .05. We are, therefore, aiming to recruit 70 patients per group to allow for dropouts.

### KARMA power calculations and sample size

KARMA was planned as a descriptive study of the changes in CKD-MBD parameters after kidney donation based on the potential number of participants available at the local center. The sample size determined was 33 kidney donors with 33 matched controls and a further comparison group consisting of 33 general population controls.
